# Clinical outcomes after vascular reconstruction using synthetic grafts for limb salvage in patients with lower extremity sarcoma: a single-center retrospective experience

**DOI:** 10.3389/fonc.2023.1199556

**Published:** 2023-08-17

**Authors:** Chuanxi Zheng, Gang Xu, Wei Li, Xin Weng, Hongwei Yang, Zuhui Wang, Shiquan Zhang

**Affiliations:** ^1^ Department of Musculoskeletal Tumor Surgery, Shenzhen Second People’s Hospital, The First Affiliated Hospital of Shenzhen University, Shenzhen, China; ^2^ Department of Pathology, Shenzhen Second People’s Hospital, The First Affiliated Hospital of Shenzhen University, Shenzhen, China; ^3^ Department of Vascular Surgery, Shenzhen Second People’s Hospital, The First Affiliated Hospital of Shenzhen University, Shenzhen, China

**Keywords:** vascular reconstruction, limb salvage, extremities sarcoma, survival, sarcoma

## Abstract

**Introduction:**

Limb-salvage surgery has become the mainstream approaches for the treatment of sarcoma in the lower extremity. In cases where the sarcoma infiltrates the primary vessel, concurrent resection of the vessels and vascular reconstruction are required to ensure sufficient resection and preservation of limb function. The objective of this study is to assess the clinical outcomes of patients who underwent vascular reconstruction utilizing synthetic grafts for limb salvage, specifically in terms of postoperative complications and limb functional status.

**Methods:**

Between September 2016 and October 2021, 15 consecutive patients who underwent 15 arterial and 3 venous reconstruction procedures were included in this retrospective study. Incidence of postoperative morbidity, graft patency, rate of limb salvage, and overall survival of patients were analyzed.

**Results:**

The median follow-up was 12.5 months (range, 4.5-72.0). Graft thrombosis occurred in 5 patients (33.3%) and graft occlusion occurred in 3 patients (20.0%). The median overall survival was 28.0 months with the estimated 2-year and 5-year overall survival of 57.8% and 43.4% respectively. The 1-year and 2-year estimated patency rates of arterial reconstructions were 82.3% and 62.1%, respectively. None of the included patients with limb amputation were observed as a consequence of severe vascular complications, while two patients underwent amputation due to the repeat recurrence, resulting in a limb salvage rate of 86.7%.

**Conclusion:**

Our results show that the combination of vascular reconstruction and oncologic resection is a feasible option for preserving limbs in cases of musculoskeletal sarcoma with vessel involvement in the lower extremity. When vascular reconstruction surgery is performed, synthetic substitutes can be effectively used with low perioperative morbidity and an acceptable rate of limb salvage.

## 1 Introduction

Sarcomas are aggressive malignant tumors of mesenchymal origin that can occur at any anatomical region of the body (1). The limbs represent the most common site of musculoskeletal sarcomas, and the lower extremity is more likely to be affected which accounts for 75% of all appendicular sarcomas (2). When sarcomas invade the major vessels, surgeons face a challenging decision to balance oncological considerations with functional preservation. Historically, sarcoma invasion of vascular structures was deemed a contraindication for limb salvage surgery, and primary amputation was the curative treatment for a complete oncologic resection (3). However, life-long morbidity and poor quality of life induced by amputation were particularly problematic.

Advances in oncology and vascular surgery have revolutionized the treatment of extremity sarcomas from amputation to limb-salvage surgery combined with vascular reconstruction (4). Reportedly, there is no significant difference in long-term survival outcomes of patients who underwent amputation or limb-salvage surgery with an adequate surgical margin (5, 6). Therefore, limb-salvage surgery has become the preferred option to preserve limb function and enhance the quality of life for patients. However, vascular reconstruction followed by oncologic resection has been reported to increase postoperative complications, which may limit the functionality of the remaining limb or result in subsequent amputation (7).

Currently, despite the minimal controversy surrounding the requirement for arterial reconstruction following the extensive tumor resection, a consensus has not yet been established regarding the optimal use of vascular substitute for the reconstruction (8). As previously reported in the literature, limb preservation rate of patients with the obstructive arterial disease could be influenced by graft selection and autologous graft has superiority to synthetic graft in terms of patency (9, 10). Furthermore, a few studies with a limited number of patients have indicated that the utilization of saphenous veins may result in decreased rates of wound dehiscence and infection in comparison to synthetic materials (11, 12). Conversely, other studies have shown no significant disparities in long-term patency and perioperative complications between patients receiving autologous or synthetic grafts (13). The influence of graft materials selection on limb-salvage rate and clinical outcomes for patients with musculoskeletal sarcomas remains controversial based on the current data set. Therefore, we retrospectively reviewed the patients with extremity musculoskeletal sarcomas who underwent vascular reconstructions using synthetic graft for limb salvage at our institution. The purpose of this study was to assess the clinical outcomes of patients following vascular reconstruction with respect to overall survival, limb function, patency of the synthetic graft, and associated postoperative complications.

## 2 Patients and methods

### 2.1 Patients selection

Institutional review board approval was acquired for this study to conduct a retrospective review of patients with musculoskeletal sarcomas of the lower extremities, who underwent a concomitant tumor resection and vascular reconstruction at the first affiliated hospital of Shenzhen university from September 1, 2016, to October 31, 2021. Due to the retrospective nature of this study with deidentified patient information, the informed consent is not necessary.

Subjects eligible for this study were those patients with musculoskeletal sarcomas in the lower extremity who required resection of the vital artery to obtain extensive surgical excision of the tumor and received the vascular reconstruction with synthetic grafts. Patients were excluded if they had malignancies of vascular origin or metastatic tumor involving the bone and soft tissue; patients with distant metastasis at initial diagnosis were also eliminated. Eventually, 15 patients met the eligibility criteria and were included in the study. Data on patient demographics, tumor site, tumor size, reconstruction surgery, functional outcomes of limb, postoperative complications and overall survival were retrospectively reviewed from medical records or telephone follow-up. A repeat review of the pathology slides of the patients was undertaken by a single sarcoma dedicated pathologist (Xin Weng), to assess the tumor histology and surgical margins.

### 2.2 Preoperative workup

All patients were initially assessed by orthopedic oncology surgeons, and computed tomography (CT) or magnetic resonance imaging (MRI) was preoperatively performed to evaluate tumor invasion of bone and major neurovascular bundle, respectively. In the event of suspected vascular invasions, computed tomography angiography (CTA) of the involved limb was performed to identify the arterial encasement. The relationship between the tumor and the major vessels was classified as described by Lucia Verga et al. (14): Type 1: the distance between the tumor and the major vessel was more than 1 cm; Type 2: the tumor was closed to the major vessel with an apparent interposition of the adipose film; Type 3: tumor adjacent to the major vessel without evident interposition of the adipose film; Type 4a: the major vessel was partially encased by the tumor; Type 4b: the major vessel was totally encased by the tumor.

In general, the treatment protocol of patients was formulated by a multidisciplinary team, which included orthopedic oncologists, vascular surgeons, radiologists, pathologists, and medical oncologists. Our institutional practice was that patients with osteosarcoma and synovial sarcoma were absolute indications for neo-adjuvant chemotherapy, myxoid liposarcoma, leiomyosarcoma, and undifferentiated pleomorphic sarcoma were relative indications for neo-adjuvant chemotherapy based on tumor grade, tumor size, and location. After the neo-adjuvant chemotherapy, the feasibility of tumor resection with sufficient margin and reconstruction strategy were discussed again according to the histologic type and the nearby structures.

### 2.3 Surgical procedure

**Figure 1:**
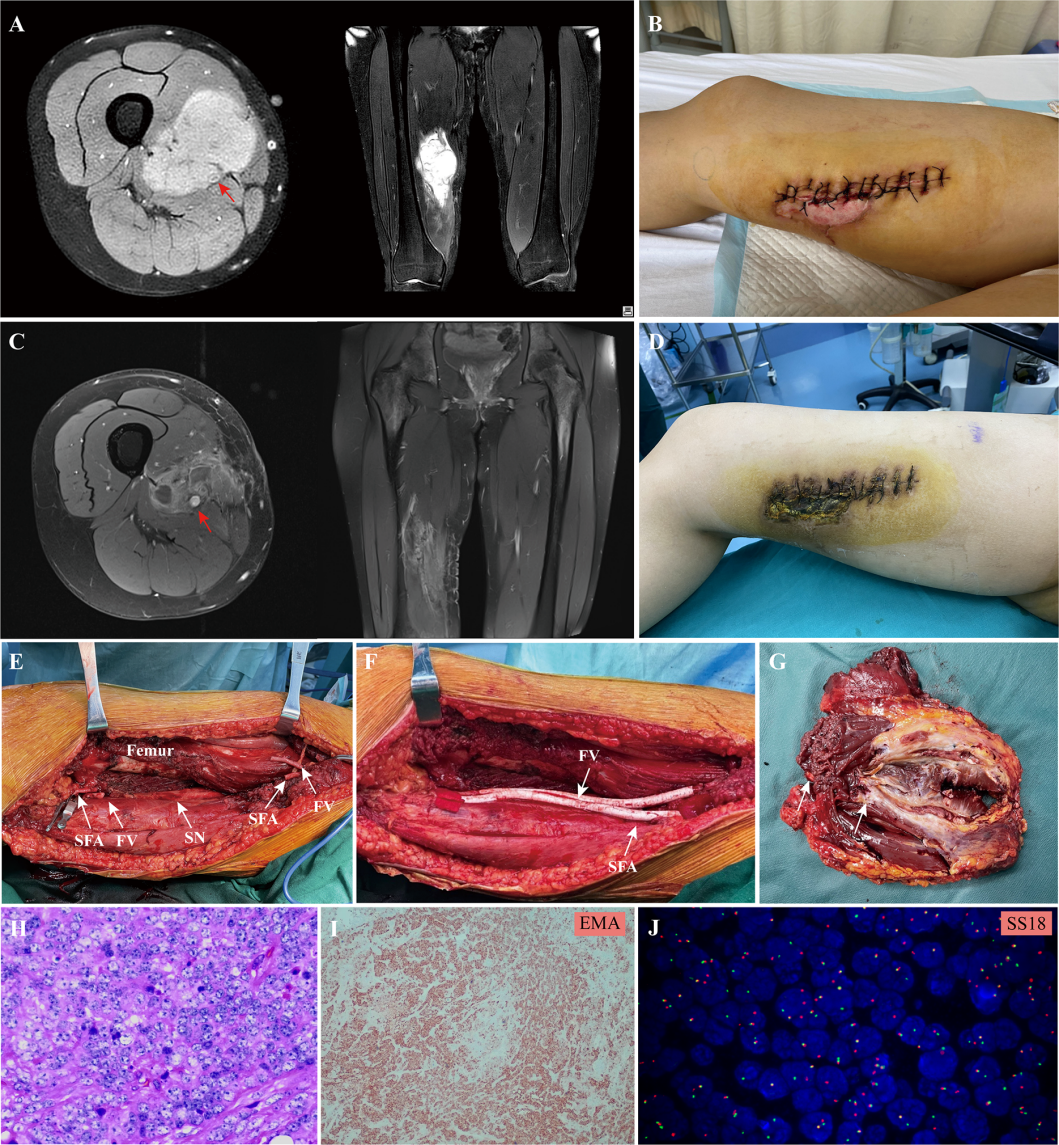
**A** 20 -year-old female patient with synovial sarcoma in the lower extremity: **(A)**, preoperative MRI showed the tumor involved the femoral vessels (red arrow) in the lower extremity; **(B)**, an unplanned resection of the tumor was performed before referral; **(C, D)**, two cycle of the adjuvant chemotherapy consisting of doxorubicin and ifosfamide was administrated, the MRI showed the size of tumor shrank after chemotherapy, but the superficial femoral artery (red arrow) and femoral vein were completely encased by the tumor; **(E)**, the extensive resection of the tumor and involved vessels were performed and **(F)** the superficial femoral artery and vein was replaced with a synthetic graft; **(G)**, the surgical margin of the resected tumor mass and the involved vessels; **(H)**, hematoxylin eosin staining and **(I)** EMA immunohistochemical staining of the tumor tissue; **(J)**, the characteristic translocation of *SS18* gene was identified by FISH; SFA, superficial femoral artery; FV, femoral vein; SN, sciatic nerve.

In this study, all patients underwent tumor removal, vascular reconstruction with bypass grafting, and skeletal reconstruction by orthopedic oncology surgeons in conjunction with vascular surgeons (Hongwei Yang and Zuhui Wang). Under general anesthesia, surgery was initially performed by orthopedic oncology surgeons to expose the tumor. Prior to the complete tumor excision, the dissection was completed around the tumor with at least 3 cm of the adjacent normal structure and vascular pedicles in situ. The intraoperative resection extent of the vessel was decided based on preoperative imaging or compression by the tumor. After an intravenous injection of heparin (5000 IU), vascular clamps were applied to the vascular pedicles by the vascular surgeon. Subsequently, the residual vessels were reconstructed utilizing a Gore^®^ PROPATEN vascular graft (W. L. Gore & Associates, Flagstaff, AZ, USA), which was composed of an expanded polytetrafluoroethylene (ePTFE) material with bound heparin to the graft surface. Depending on the diameter and size of the resected vessel, a synthetic graft with a suitable diameter was used for vascular reconstructions. Following the arterial end‐to‐end anastomosis, the blood supply of the lower limb and distal flow was intraoperatively evaluated through pulse palpation at the dorsal pedal artery. Reconstruction of the major venous system was indicated when the collateral venous outflow of the lower extremity was predominately blocked by thrombotic occlusion or resected along with the tumor. Subsequently, the orthopedic oncology surgeon reconstructed any segmental bone defect with modular endoprosthesis, if deemed necessary, and closed the wound over suction drains. A representative case of synovial sarcoma in the lower extremity after an unplanned resection was shown in **Figure 1**.

### 2.4 Postoperative management

Following surgery, thromboprophylaxis and prophylactic antibiotics were administered as standard practice. Patients without a risk of bleeding were given subcutaneous enoxaparin (40mg/QD) for anticoagulation therapy until oral administration was feasible. Additionally, antiplatelet therapy was continued for a minimum of six months. Postoperative adjuvant therapies were determined by our multidisciplinary team, with chemotherapy prescribed for sensitive tumors and local radiotherapy for tumors relatively insensitive to chemotherapy.

The patients were assessed in the vascular and orthopedic outpatient clinic monthly for the first three months, every three months for the first two years, and every six months thereafter. Imaging studies including color flow duplex scan, CT chest and MRI were performed at each follow-up visit to evaluate the vascular graft patency, local tumor control and distant metastasis. Physical examination including capillary filling and distal artery palpation were recorded to evaluate the distal vascular supply. Functional outcomes of the lower extremities were measured according to the Musculoskeletal Tumor Society (MSTS) 93 score system, which is based on six items: pain, overall function, emotional acceptance, support, walking ability, and gait (15).

### 2.5 Statistical analysis

All statistical analysis of this study was carried out using STATA Statistical Software (version 16, College Station, TX, USA). Descriptive statistics of mean and standard deviation or range were used for continuous variables and categorical variables (number and percentage). A chi-square test (or Fisher exact test) was run to assess the differences between categorical variables, and an unpaired Student t-test was used for continuous variables. The probability of patient survival and the rates of vascular graft patency were estimated according to the Kaplan-Meier method. A two-tailed P-value <0.05 was considered statistically significant.

## 3 Results

### 3.1 Patients’ characteristics

During the study period, a total of 15 patients who underwent sarcoma resection combined with vascular reconstruction using synthetic grafts were included in this retrospective study. The demographics, clinical characteristics, preoperative and postoperative treatment of patients were summarized in **Table 1**. The mean age of the patients was 36.0 (range 15-68) years, including 10 males and 5 females. Histological diagnoses included osteosarcoma (n=5, 33.3%), synovial sarcoma (n=3, 20.0%), undifferentiated pleomorphic sarcoma (n=3, 20.0%), liposarcoma (n=3, 20.0%), and leiomyosarcoma (n=1, 6.7%). According to the Musculoskeletal Tumor Society Staging System, there was one patient (6.7%) with stage IB, 12 patients (80.0%) with stage IIB, and two patients (13.3%) with stage III sarcoma. The tumor originated from the groin regions in four patients (26.7%), the thigh in seven patients (46.7%), the popliteal region in three patients (20.0%), and the crus in one patient (6.7%). Of those, 11 (73.3%) of the tumors treated were primary sarcomas, and four (26.7%) were recurrent sarcomas. The majority of patients had received perioperative adjuvant therapy, except for three patients (20.0%) who did not receive any systemic or local therapy prior to treatment; 11 patients (73.3%) underwent neoadjuvant chemotherapy, but one patient (6.7%) with myxoid liposarcoma received preoperative radiotherapy. Prior to surgery, five patients diagnosed with osteosarcoma underwent a minimum of two cycles of neoadjuvant chemotherapy comprising doxorubicin, cisplatin, and ifosfamide. In addition, six patients with soft tissue sarcoma received a combination chemotherapy regimen with doxorubicin and ifosfamide for two cycles. Postoperatively, 12 patients (80%) were subjected to adjuvant chemotherapy based on the histologic type, while three patients (20%) received the radiotherapy due to the inability to tolerate chemotherapy.

**Table 1:** Patient demographics and clinical characteristics.

Characteristics		Patients (%)
Age		
	Mean	36.0
	Range	15-68
Gender		
	Female	5 (33.3%)
	Male	10 (66.7%)
Histologic type		
	Liposarcoma	3 (20.0%)
	Synovial sarcoma	3 (20.0%)
	Undifferentiated pleomorphic sarcoma	3 (20.0%)
	Leiomyosarcoma	1 (6.7%)
	Osteosarcoma	5 (33.3%)
Enneking stage		
	I B	1 (6.7%)
	II B	12 (80.0%)
	III	2 (13.3%)
Tumor site		
	Groin regions	4 (26.7%)
	Thigh regions	7 (46.7%)
	Popliteal region	3 (20.0%)
	Lower leg	1 (6.7%)
Tumor size		
	≤10 cm	5 (33.3%)
	>10 cm	10 (66.7%)
Vessels involved		
	Common femoral artery	2 (13.3%)
	Superficial femoral artery	8 (53.3%)
	Popliteal artery	4 (26.7%)
	Post-tibial artery	1 (6.7%)
Previous treatment		
	Primary tumor	11 (73.3%)
	Recurred tumor	4 (26.7%)
Preoperative therapy		
	Neoadjuvant chemotherapy ^a^	11 (73.3%)
	Radiation	1 (6.7%)
	None	3 (20.0%)
Postoperative therapy		
	Adjuvant chemotherapy	12 (80.0%)
	Radiation	3 (20.0%)

a, the neoadjuvant chemotherapy regimens for osteosarcoma were included doxorubicin, cisplatin and ifosfamide; the neoadjuvant chemotherapy regimens for soft tissue sarcoma were included doxorubicin and ifosfamide.

### 3.2 Reconstruction

Patient clinical characteristics, the vessels involvement and vascular reconstruction type were summarized in **Table 2**. The mean diameter of the tumor was 12.5 cm (range 6.0-33.0 cm). With respect to the involvement of major vessels, sarcoma partially encased major vessels (Type 4a) in 6 out of 15 cases (40.0%), while total encasement (Type 4b) was observed in nine patients (60.0%). The most frequently performed vascular reconstruction was femoral-femoral bypass in eight patients (53.3%), followed by femoral-popliteal bypass in four patients (26.7%). In this study, other reconstructions also were performed, including common femoral to the superficial femoral artery in two patients (13.3%) and popliteal to post-tibial artery in one patient (6.7%). Venous reconstruction was indicated only when both the greater saphenous vein and femoral deep vein were impaired during oncologic resection. In total, 18 vessels were reconstructed in 15 patients, of which three patients underwent both arterial and venous reconstructions, while the remaining 12 patients received solely arterial reconstructions and venous ligation after tumor resection. Notably, all patients achieved complete resection of the tumor with a microscopic negative margin (R0 resection), although marginal resection with a narrow margin of less than 3 cm was observed in two patients. Six patients (40.0%) underwent extensive tumor resection followed by skeletal reconstruction using modular endoprosthesis, of which one patient with tibial osteosarcoma received a gastrocnemius flap transposition to cover the endoprosthesis. The remaining nine patients (60.0%) were treated with wide resection and soft tissue reconstruction.

**Table 2:** Preoperative characteristics of patients and vascular reconstruction surgery performed.

Patient	Age	Gender	Diagnosis	Tumor site	Stage	Size (cm)	Vessels Type	Chemotherapy Preop/postop	Radiotherapy Preop/postop	Vessels Reconstructed	Surgery
1	29	Female	OS	Popliteal	II B	6	4b	Neo/Adjuvant	–	PA	Endoprosthesis Replacement after resection
2	42	Male	LS	Thigh	II B	11	4a	–	-/Adjuvant	SFA	Endoprosthesis Replacement after resection
3	30	Male	OS	Popliteal	II B	7	4b	Neo/Adjuvant	–	PA	Endoprosthesis Replacement after resection
4	63	Male	UPS	Groin	III	13	4a	Neo/-	-/Adjuvant	SFA	Resection
5	21	Male	OS	Popliteal	II B	14	4b	Neo/Adjuvant	–	PA/PV	Endoprosthesis Replacement after resection
6	15	Male	OS	Thigh	III	11	4b	Neo/Adjuvant	–	PA	Endoprosthesis Replacement after resection
7	20	Female	SS	Thigh	II B	9	4b	Neo/Adjuvant	–	SFA/SFV	Resection
8	27	Male	OS	Crus	II B	10	4b	Neo/Adjuvant	–	PTA	Endoprosthesis Replacement after resection
9	32	Male	LS	Thigh	I B	33	4a	-/Adjuvant	Neo/-	SFA	Resection
10	44	Female	SS	Groin	II B	7	4b	Neo/Adjuvant	–	SFA	Resection
11	52	Female	LMS	Thigh	II B	12	4a	-/Adjuvant	–	SFA	Resection
12	68	Female	UPS	Thigh	II B	13	4a	–	-/Adjuvant	SFA	Resection
13	31	Male	SS	Groin	II B	14	4b	Neo/Adjuvant	–	CFA	Resection
14	35	Male	UPS	Groin	II B	17	4b	Neo/Adjuvant	–	CFA	Resection
15	25	Male	LS	Thigh	II B	11	4a	Neo/Adjuvant	–	SFA/SFV	Resection

OS, osteosarcoma; LS, liposarcoma; UPS, Undifferentiated pleomorphic sarcoma; SS, Synovial sarcoma, LMS, Leiomyosarcoma; Preop, Preoperative; Postop, Postoperative; Neo, Neoadjuvant; PA, Popliteal artery; SFA, superficial femoral artery; PV, Popliteal vein; SFV, superficial femoral vein; PTA, Popliteal tibial artery; CFA, common femoral artery.

### 3.3 Complications

Early postoperative limb edema was the most frequent complication, which was observed in eight patients (53.3%), and conservatively managed by elevation of the limb and use of elastic support. Wound dehiscence and delayed wound healing occurred in two patients (13.3%), and all these patients necessitate additional surgical interventions to achieve wound healing. A wound minor hematoma developed in one patient (6.7%) with an extremely large tumor located in the thigh, which was successfully treated by temporary discontinuation of oral antiplatelets therapy and local compression. None of the included patients encountered acute arterial graft thrombosis, wound infection, or nerve palsy.

During the follow-up period, five patients (33.3%) experienced graft thrombosis. Of these patients, two patients had superficial femoral artery thrombosis and three patients had popliteal artery thrombosis after surgery. None of the patients exhibited any overt clinical symptoms of subacute ischemia in the lower limb, and no further occlusion was observed after anticoagulation treatment. However, one patient with popliteal arterial and venous reconstruction encountered venous occlusion at 8 months after surgery. Two patients who underwent arterial reconstruction experienced graft occlusions at 9 and 11 months postoperatively, respectively. Conservative treatment was administered to all patients with vascular occlusion, as they exhibited collateral circulation to the distal limb and no signs of extremity ischemia. No instances of limb amputation were observed as a result of severe vascular complications. However, two patients underwent amputation due to local disease recurrence following the initial operation. One patient diagnosed with femoral osteosarcoma underwent an initial endoprosthesis replacement following tumor resection. Despite the absence of distant metastasis, the patient had repeated local recurrence, resulting in severe pain and ambulatory difficulties. Consequently, the patient underwent hip disarticulation to alleviate symptoms and improve quality of life. Another patient with tibial osteosarcoma developed local recurrence and bone metastasis in the ipsilateral foot after re-excision surgery. Despite receiving amputation with hip disarticulation, the patient ultimately succumbed to complications arising from multiple lung metastases.

### 3.4 Survival and functional outcome

**Figure 2:**
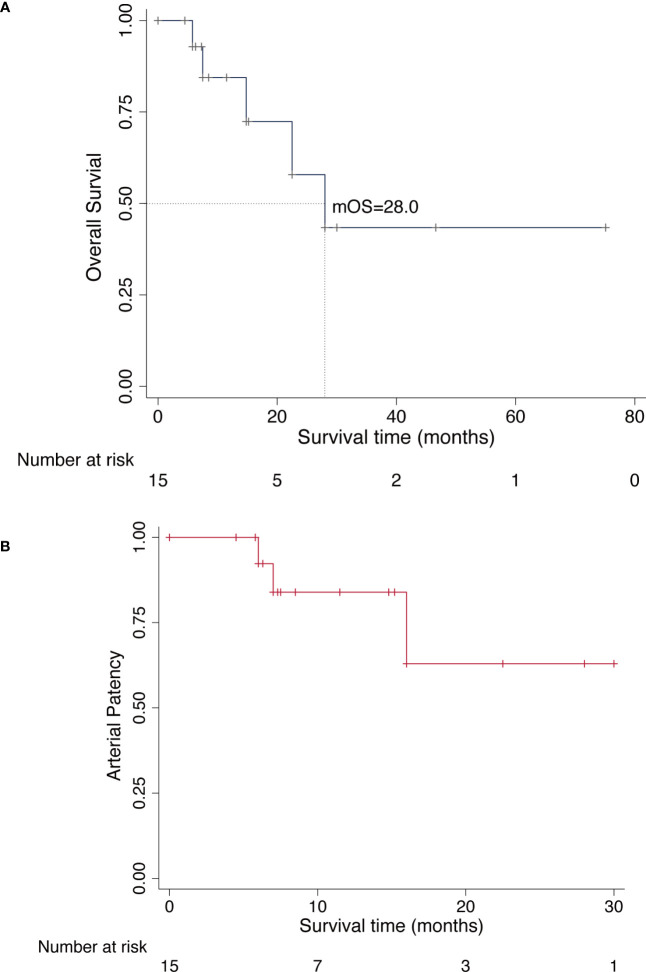
Kaplan-Meier curves for overall survival and arterial patency. **(A)**, Overall survival curve of all patients who underwent limb-sparing surgery for lower extremity sarcoma with vascular reconstruction. **(B)**, Overall patency curve of all patients who underwent arterial reconstruction.

In the last analysis, the median follow-up was 12.5 months with a range from 4.5 to 72.0 months. The local recurrence occurred in three patients (20.0%) throughout the follow-up period and one patient died of disease after 14.8 months who developed multiple lung metastases without any further treatment; two patients underwent wide local re-excision, but they underwent amputation surgery as a result of re-recurrence after re-excision surgery; Pulmonary metastasis developed in other four patients. Although these patients received adjuvant chemotherapy or targeted therapy (tyrosine kinase inhibitors), all patients ultimately died of the disease (**Table 3**). At the last follow-up visit, 10 patients (66.7%) were alive without evidence of disease, and five patients (33.3%) were dead of disease. Among these 15 patients, 13 patients (86.7%) had successful limb salvage and no limb amputations were caused by vascular complications. The median survival was 28.0 months (95% CI, 7.5-39.2), and the estimated 2-year and 5-year overall survival of our patients was 57.8% (95% CI, 20.4%-82.8%) and 43.4% (95% CI, 10.9%-72.9%), respectively, as shown in **Figure 2A**. Concerning the patency of vascular graft, the 1-year and 2-year patency rates of arterial reconstructions were 82.8% (95% CI, 46.7%-95.4%) and 62.1% (95% CI, 19.7%-87.9%), respectively, as shown in **Figure 2B**. The postoperative functional status of patients requiring vascular reconstruction was evaluated with the MSTS score. The results indicated a mean MSTS score of 24.9, ranging from 22 to 28. Of these patients, 11 patients (73.3%) exhibited satisfactory function (MSTS score ≥ 24) in the involved limb, and none of the surviving patients required walking aids or crutches during the final follow-up. One patient with endoprosthesis reconstruction complained of pain in the thigh when walking unsupported for distances longer than 3,000 m, but had no radiographic evidence of prosthesis loosening and other prosthetic complications. Neither pain nor gait abnormalities were noted in the other patients at the recent follow-up.

**Table 3:** Postoperative complications, functional and survival outcome of patients during the follow‐up period.

Patient	Age	Gender	Diagnosis	Tumor site	Vessels Type	Vessels Reconstructed	Resection margin	Complications	Survival	MSTS score	Limb Salvage	Follow-up (m)
1	29	Female	OS	Popliteal	4b	PA	Wide	Occlusion, Recurrence	NED	27	No	72.0
2	42	Male	LS	Thigh	4a	SFA	Wide	Edema, Thrombosis, Wound disentrance	NED	23	Yes	46.6
3	30	Male	OS	Popliteal	4b	PA	Wide	Thrombosis, Metastasis	DOD	25	Yes	22.5
4	63	Male	UPS	Groin	4a	SFA	Wide	Edema, Metastasis	DOD	28	Yes	18.5
5	21	Male	OS	Popliteal	4b	PA/PV	Marginal	Thrombosis, Occlusion, Recurrence, Metastasis	DOD	23	Yes	14.8
6	15	Male	OS	Thigh	4b	PA	Wide	Edema, Thrombosis	NED	24	Yes	30.0
7	20	Female	SS	Thigh	4b	SFA/SFV	Wide	–	NED	27	Yes	7.3
8	27	Male	OS	Crus	4b	PTA	Wide	Recurrence	NED	24	No	8.5
9	32	Male	LS	Thigh	4a	SFA	Marginal	Edema, Wound minor hematoma	NED	22	Yes	11.5
10	44	Female	SS	Groin	4b	SFA	Wide	Edema, Thrombosis, Wound disentrance	NED	25	Yes	15.2
11	52	Female	LMS	Thigh	4a	SFA	Wide	Edema, Occlusion, Metastasis	DOD	26	Yes	12.5
12	68	Female	UPS	Thigh	4a	SFA	Wide	–	NED	25	Yes	5.8
13	31	Male	SS	Groin	4b	CFA	Wide	Edema, Metastasis	DOD	22	Yes	9.5
14	35	Male	UPS	Groin	4b	CFA	Wide	Edema	NED	26	Yes	4.5
15	25	Male	LS	Thigh	4a	SFA/SFV	Wide	–	NED	27	Yes	7.5

OS, osteosarcoma; LS, liposarcoma; UPS, Undifferentiated pleomorphic sarcoma; SS, Synovial sarcoma, LMS, Leiomyosarcoma; PA, Popliteal artery; SFA, superficial femoral artery; PV, Popliteal vein; SFV, superficial femoral vein; PTA, Popliteal tibial artery; CFA, common femoral artery. DOD: Dead of disease, NED: No evidence of disease.

## 4 Discussion

Surgery represents the mainstay treatment for extremity musculoskeletal sarcomas, and local tumor control is a critical predictor determining the prognosis of patients (16). Although the curative amputation was commonly performed in patients with sarcomas involving the major vascular bundle of extremities in the past, the contemporary approaches prioritize limb salvage and have yielded improved patient outcomes (17). It is noteworthy that limb preservation with sufficient margins is comparable to amputation in terms of disease-free and overall survival rates (18). Whereas, to achieve negative margins of tumor resection, an inevitable resection of the vascular bundle and concomitant vascular reconstruction were necessitated when major vessels were invaded by the tumor.

Most current reconstructive techniques involve using autologous or synthetic grafts to interpose the vessel defects rather than vascular anastomosis, owing to extensive resection of the tumor frequently requiring the excision of a long segment of vessels. In general, reconstruction with autologous grafts seems to be preferable to synthetic grafts, since autologous grafts had superior vascular patency rates than synthetic grafts (11, 13). Moreover, another important advantage of the autologous graft was to reduce the requirement for long-term anticoagulation therapy. Nevertheless, the shortcomings of autologous graft include: it is vulnerable to kinking and compression at the groin area or popliteal fossa, where is the flexion place of the hip and knee joint (19); the optimal autogenous graft is not readily available concerning the suitable diameter of the arterial stump and the required length of vessels defect; the excess surgical wounds for harvesting autogenous graft also cause secondary morbidity and may increase risk of surgical complications. On the other hand, the previous studies demonstrated vascular reconstruction with the synthetic graft can be effectively used without increased risk of postoperative complications. Therefore, the choice of vascular substitute for the reconstruction of the residual vessels in limb-salvage surgery is controversial (20, 21).

In the present study, 15 patients who required vascular reconstruction following wide resection of sarcoma in the lower extremity between 2016 to 2021 were included. Totally, five patients developed graft thrombosis, but all patients with arterial thrombosis were managed conservatively without further morbidity. Furthermore, graft occlusion was noted in two patients with arterial reconstruction and one patient with venous reconstruction, while no critical ischemia symptom or venous return insufficiency was found in the lower extremity. This suggests that more extensive collateral circulation including artery and vein has formed prior to graft occlusion, thereby providing adequate blood supply to the distal extremity. No patients underwent limb amputation as a result of severe vascular complications. Regarding the patency of the synthetic graft, the overall primary patency rate at one year and two-year was 82.8% and 62.1%, respectively. According to other studies that report graft patency of synthetic grafts in the lower limb ranging from 65% to 75%, the one-year patency rates in the present study are comparable (20, 21). Although the arterial patency rates after reconstructions with autologous grafts in the lower limb reported by other studies were around 90%, a majority of the cited studies did not definitively discriminate the primary and primary assisted patency (7, 13, 22). Wortmann and colleagues have reported the one-year primary patency of arterial reconstructions after the resection of tumor in the lower extremity was 81%, but the two-year patency decreased to 65% (20). With the assistance of the additional procedure, the overall patency rates of autologous grafts at postoperative one and two years were 88% and 72%, respectively. However, comparable to the autologous graft, the selection of the bypass material for the resected vessels did not significantly impact the primary or primary assisted patency (20). Additionally, Mlees and colleagues reported that there was no significant difference between the synthetic graft and autologous graft with respect to wound infection, wound dehiscence and vascular consequences (21).

Currently, the optimal management of the venous system after resection is debatable. While many authors recommend routine venous reconstruction to alleviate limb swelling and chronic venous insufficiency resulting from deep venous obstruction (23, 24), others advocated venous ligation and denied any benefit of venous reconstruction due to the low rate of long-term patency and high rate of complications (7, 22). Our institutional protocol for venous reconstruction entails the concurrent resection of the great saphenous vein, femoral vein, and venous collaterals in the adductor muscles, as the elimination of these venous collaterals may lead to severe sequelae and venous disease (20). To compensate venous backflow, it is crucial to preserve the superficial fascia veins when tumors invade the deep venous system. In the present study, the majority of veins were ligated during tumor resection without reconstruction, with only three patients (20.0%) undergoing venous reconstruction. Although limb swelling and edema were the most frequent postoperative complication in our series, they did not require any further intervention and were well managed with conservative treatment. We speculated that the early limb swelling after surgery is probably attributed to the extensive excision of soft tissues and the resultant damage to lymphatic vessels instead of a pure insufficiency return of the venous system. In accordance with previous reports, patients without venous reconstruction did not encounter any advanced venous disease during the follow-up period (11, 25). Thus, our results provide additional evidence that the ligation of veins in conjunction with extensive resection of musculoskeletal sarcoma in the lower limb is both safe and feasible (20, 26).

Recent research concerning oncological outcomes after limb-sparing surgery has depicted a low recurrence rate after extensive resection of musculoskeletal sarcoma (27). As demonstrated by previous studies, the survival rates of patients who underwent vascular reconstruction after wide resection of sarcoma was comparable to those of patients without vascular involvement (7). For this reason, the need for vascular resection and reconstruction should not be a deterrent to extensive resection for patients with extremity sarcomas encasing major vessels. In our series, there were three patients (20.0%), who received limb-sparing surgery and experienced a local recurrence. Two of these three patients underwent limb amputation owing to local tumor recurrence after re-excision surgery. The limb salvage rate was 86.7% at the time of the last follow-up, which is comparable with previous studies about autograft reconstruction (8). During the follow-up period, 11 of 15 patients (73.3%) achieved an excellent or good function of the involved limb. The functional assessments of limb show that motion and stability are mostly excellent or good with a mean MSTS score of 24.9. Therefore, a limb salvage procedure provided patients with satisfactory limb function, and contribute to the improvement of life quality.

Metastatic disease is a significant concern for malignant tumors, as it often results in decreased survival rates even after local tumor control. Radaelli et al. reported the clinical outcome of 113 patients who received vascular reconstructions after soft tissue sarcoma resections (22). Among these patients, distant metastasis occurred in more than 50% of patients at a median follow-up of 3 years (22). Overall, 2 and 5-year survivals in patients who underwent vascular reconstruction followed by the sarcoma resection have been reported to range between 40.2% to 77.7% and 37.7% to 52.0%, respectively, according to the anatomical site and surgical margin (28, 29). In our series, distant metastases were observed in five patients (33.3%), including two of five patients with osteosarcoma and 3 of 10 patients with soft tissue sarcoma. The 57.8% overall estimated 2-year survival and 43.4% overall estimated 5-year survival for our cohort were comparable to the data reported by previous literature. Noteworthy, five of nine patients (55.6%) with sarcoma completely enclosing the vessels (Type 4b), while two of six patients (33.3%) with sarcoma partially enclosing the vessels (Type 4a) experienced postoperative recurrence or distant metastasis during the follow-up. According to the previous studies, microscopic vascular invasion by malignant tumors was associated with an increased risk of metastasis and a poor prognosis (30, 31). Patients with total encasement of the vessels (Type 4b) were more frequently associated with vascular displacement and stenosis, which indicates a high risk of encroachment of the vessels at surgery (32). Poultsides et al. conducted a study involving 34 patients with primary musculoskeletal sarcoma that involved major vessels in the extremities. The results demonstrated that 43% of patients with extremity sarcoma exhibited histologically confirmed tumor invasion of the resected vessel wall (7). While the author did not distinguish the type of vessels involvement (partial or complete), it is plausible that patients with complete encasement of the vessels may have a greater risk of genuine vascular invasion compared to those with alternative patterns of vessel involvement. However, further validation in additional studies with large sample sizes is warranted.

Additionally, our study has several important limitations: this study had a retrospective design without a comparison group, not all possible clinical data could be reliably retrieved and might be affected by review bias. The small sample size of patients with heterogeneous diagnoses and a brief follow-up period may restrict the generalizability of our findings. The different histologic types of tumors may have a direct impact on treatment plans and prognosis. Furthermore, the efficacy of adjuvant chemotherapy or radiotherapy on survival for patients treated at other facilities remains uncertain. Further multicenter researches that include larger samples, longer-term follow-up, and randomization are necessary to validate the optimal vascular substitute for patients requiring vascular reconstruction during limb-salvage surgery for lower extremity sarcomas.

## 5 Conclusion

Musculoskeletal sarcomas with involvement of major blood vessels are not a deterrent to limb-salvage surgery, vascular reconstruction combined with oncological surgery is feasible to provide a favorable limb function and improved life quality of patients. When vascular reconstruction surgery is performed, the synthetic substitute is an alternative option with accepted outcomes in terms of postoperative complications and rate of limb salvage.

## Data availability statement

The original contributions presented in the study are included in the article/supplementary material. Further inquiries can be directed to the corresponding author.

## Ethics statement

This study was reviewed and approved by Biological and Medical Ethics Committee of the First Affiliated Hospital of Shenzhen University.

## Author contributions

Conceptualization, CZ, GX; methodology, CZ, GX; writing-original draft preparation, CZ, GX; resources, HY, XW, ZW; data curation WL, SZ; writing-review and editing, WL, SZ; supervision, SZ. All authors have read and agreed to the published version of the manuscript and were responsible for the concept of this paper and drafting the manuscript.
